# QTL detection and candidate gene analysis of grape white rot resistance by interspecific grape (*Vitis vinifera* L. × *Vitis davidii* Foex.) crossing

**DOI:** 10.1093/hr/uhad063

**Published:** 2023-04-02

**Authors:** Peng Li, Xibei Tan, Ruitao Liu, Faiz Ur Rahman, Jianfu Jiang, Lei Sun, Xiucai Fan, Jihong Liu, Chonghuai Liu, Ying Zhang

**Affiliations:** National Key Laboratory for Germplasm Innovation and Utilization of Horticultural Crops, Zhengzhou Fruit Research Institute, Chinese Academy of Agricultural Sciences, Zhengzhou 450000, China; Key Laboratory of Horticultural Plant Biology (MOE), College of Horticulture and Forestry Sciences, Huazhong Agricultural University, Wuhan 430000, China; National Key Laboratory for Germplasm Innovation and Utilization of Horticultural Crops, Zhengzhou Fruit Research Institute, Chinese Academy of Agricultural Sciences, Zhengzhou 450000, China; National Key Laboratory for Germplasm Innovation and Utilization of Horticultural Crops, Zhengzhou Fruit Research Institute, Chinese Academy of Agricultural Sciences, Zhengzhou 450000, China; National Key Laboratory for Germplasm Innovation and Utilization of Horticultural Crops, Zhengzhou Fruit Research Institute, Chinese Academy of Agricultural Sciences, Zhengzhou 450000, China; National Key Laboratory for Germplasm Innovation and Utilization of Horticultural Crops, Zhengzhou Fruit Research Institute, Chinese Academy of Agricultural Sciences, Zhengzhou 450000, China; National Key Laboratory for Germplasm Innovation and Utilization of Horticultural Crops, Zhengzhou Fruit Research Institute, Chinese Academy of Agricultural Sciences, Zhengzhou 450000, China; National Key Laboratory for Germplasm Innovation and Utilization of Horticultural Crops, Zhengzhou Fruit Research Institute, Chinese Academy of Agricultural Sciences, Zhengzhou 450000, China; Key Laboratory of Horticultural Plant Biology (MOE), College of Horticulture and Forestry Sciences, Huazhong Agricultural University, Wuhan 430000, China; National Key Laboratory for Germplasm Innovation and Utilization of Horticultural Crops, Zhengzhou Fruit Research Institute, Chinese Academy of Agricultural Sciences, Zhengzhou 450000, China; National Key Laboratory for Germplasm Innovation and Utilization of Horticultural Crops, Zhengzhou Fruit Research Institute, Chinese Academy of Agricultural Sciences, Zhengzhou 450000, China; Zhongyuan Research Center, Chinese Academy of Agricultural Sciences

## Abstract

Grape white rot, a devastating disease of grapevines caused by *Coniella diplodiella* (Speg.) Sacc., leads to significant yield losses in grape. Breeding grape cultivars resistant to white rot is essential to reduce the regular use of chemical treatments. In recent years, Chinese grape species have gained more attention for grape breeding due to their high tolerance to various biotic and abiotic factors along with changing climatic conditions. In this study, we employed whole-genome resequencing (WGR) to genotype the parents of ‘Manicure Finger’ (*Vitis vinifera*, female) and ‘0940’ (*Vitis davidii*, male), along with 101 *F*_1_ mapping population individuals, thereby constructing a linkage genetic map. The linkage map contained 9337 single-nucleotide polymorphism (SNP) markers with an average marker distance of 0.3 cM. After 3 years of phenotypic evaluation of the progeny for white rot resistance, we confirmed one stable quantitative trait locus (QTL) for white rot resistance on chromosome 3, explaining up to 17.9% of the phenotypic variation. For this locus, we used RNA-seq to detect candidate gene expression and identified *PR1* as a candidate gene involved in white rot resistance. Finally, we demonstrated that recombinant PR1 protein could inhibit the growth of *C. diplodiella* and that overexpression of *PR1* in susceptible *V. vinifera* increased grape resistance to the pathogen.

## Introduction

**Figure 1:**
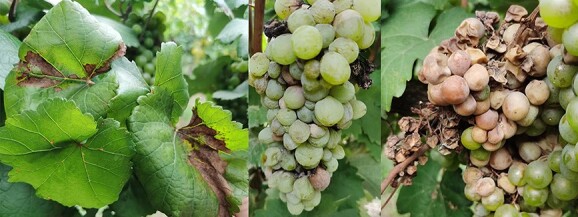
Symptoms of leaf and berry diseases caused by *C. diploeaea* infection

Grapevine is a prominent global horticulture crop that has been grown for millennia to provide fresh fruit, wine, juice, and raisins. However, the grape is subject to a variety of diseases, including grape powdery mildew, white rot, black rot, and downy mildew [1–4]. Grape white rot, a devastating disease of grapevines caused by *Coniella diplodiella* (Speg.) Sacc., attacks leaves, branches, and berries, leading to a reduction in grape output and quality [5, 6] (Fig. 1). Surveys of grape white rot in China revealed that the disease is widespread in significant grape-growing regions [7]. Despite efforts of growers to limit the occurrence of white rot through thinning, leaf removal, and pruning operations to increase air and light circulation, the disease can still manifest under unfavorable environmental conditions such as heavy rain and hail [7]. As a result, the control of grape white rot in grape production requires regular application of chemicals, which not only increases production cost but is also harmful to the environment and is against the policies of food safety measures [8]. Breeding of grape white rot disease-resistant varieties is beneficial for the healthy development of the grape industry. Traditional crossbreeding procedures are time-consuming and inefficient. Adopting the marker-assisted selection strategy, which enables the targeted selection of progeny with resistance loci, is one way to significantly expedite the breeding process [9, 10].

The study of pathogen resistance genetics involves the establishment of population phenotypes, as well as the construction of genetic maps to identify genomic regions associated with target traits and genetic markers that can be used for marker-assisted selection across different backgrounds [8]. In recent years, numerous disease-resistant loci have been discovered. For example, resistance loci for downy mildew (*Rpv1*–*Rpv31*) have been identified on various chromosomes (Chr 3, 4, 5, 6, 7, 8, 9, 11, 12, 14, 16, 17, and 18) in different genetic backgrounds [9, 11–16]. Similarly, resistance loci for powdery mildew have been discovered in Chr 2 (*Ren10*), 9 (*Ren6*), 12 (*Run1*), 13 (*Ren1*), 14 (*Ren2*, *Ren5*), 15 (*Ren3*, *Ren9*), 18 (*Run2.1*, *Run2.2*, *Ren4*, *Ren8*), and 19 (*Ren7*) [3, 9, 17–24]. Most of these resistance sources originate from Asian and North American *Vitis* species, including *V. amurensis*, *V. labrusca*, *V. riparia*, and *V. rupestris*, and *Muscadinia rotundifolia* [13–16, 25]. Nonetheless, for white rot resistance, only a single quantitative trait locus (QTL) analysis has been described. The authors identified one QTL on Chr 14 using one *V. labrusca* mapping population of ‘Zhuosexiang’ × ‘Victoria’ [25]. Compared with North American *Vitis* species, Chinese grape species have garnered more attention in grape breeding due to their fruit flavors, full inter-fertility with European grape species, and high resistance to white rot [5, 8]. *Vitis davidii*, as reported by Zhang *et al*. [6], is the most resistant Chinese *Vitis* species to grape white rot disease [6]. However, limited genetic research has been conducted on this species. The lack of a genetic map from this species impedes the efficacy of breeding efforts through marker-assisted selection and explains the genetic basis of *V. davidii*’s high resistance traits.

Plants are constantly exposed to a variety of pathogenic agents, including bacteria, fungi, oomycetes, and viruses, which pose significant challenges to their growth and development. To protect themselves, plants have evolved two types of immune response: pathogen-associated molecular pattern-triggered immunity (PTI) and effector-triggered immunity (ETI). PTI is initiated when a pattern recognition receptor (PRR) recognizes pathogen-associated molecular patterns (PAMPs) and activates the plant immune system. On the other hand, ETI is activated when resistance (R) proteins recognize pathogen effector proteins (PEPs). ETI triggers the hypersensitive response (HR), which induces cell death at the site of infection, limiting local pathogen dissemination [26–28]. Furthermore, ETI can elicit systemic transfer of immune signals, triggering the synthesis of pathogenesis-related (PR) proteins with antimicrobial activity, thereby enhancing plant defense against subsequent pathogenic assaults [28–30]. Among the PR family, pathogenesis-related 1 (PR1) proteins are essential and are produced abundantly during defense responses [31, 32]. Su *et al*. [33] demonstrated that the expression of *PR1* is regulated by crosstalk between salicylic acid and jasmonic acid, which is crucial for grapevine resistance against white rot.

In this work, we generated a linkage map using a population of ‘Manicure Finger’ (*Vitis vinifera*, susceptible) × ‘0940’ (*V. davidii*, resistant). The map, in combination with phenotypic data, was able to identify one stable white rot resistance QTL on Chr 3. According to the mapped QTL and the RNA-seq results, one potential *PR1* gene was analyzed that may be related to white rot.

## Results

### Phenotypic evaluation

**Figure 2:**
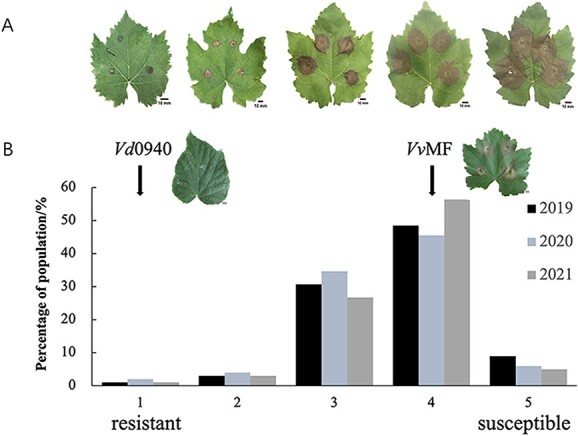
Distribution of scored variables in the *Vv*MF × *Vd*0940 mapping population. **A** Representative images of the five-point rating scale for leaves infected with *C. diplodiella*, spanning from the most resistant to the most susceptible. **B** Distribution of scores for progeny and two parents.

In July of 2019, 2020, and 2021, 101 hybrid progeny were evaluated for resistance to white rot. Their susceptibility to white rot ranges from 1 (most resistant) to 5 (most susceptible). *Vd*0940 (male) displayed a distribution of resistance at level 1, while *Vv*MF (female) showed a susceptibility distribution at level 4. Most of the offspring showed a susceptibility level of 4 (45.5–56.4%), followed by level 3 (26.7–34.7%) (Fig. 2, Supplementary Data S1). Moreover, there was a significant correlation between the grape white rot resistance scores in three years according to Pearson's correlation coefficient (*P* < .001) (Supplementary Data Fig. S1).

### Construction of genetic linkage map

**Figure 3:**
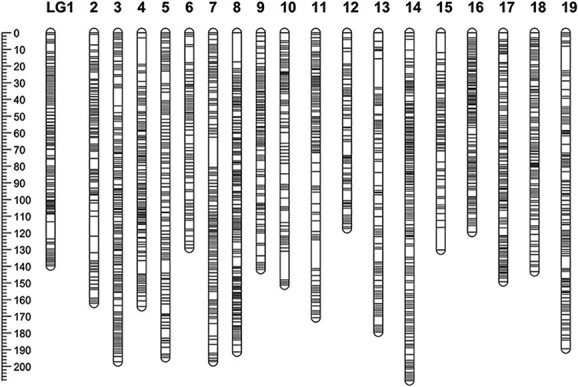
Integrated genetic map of the *Vv*MF × *Vd*0940 population with 101 individuals. The vertical scale depicts genetic length, the unit being cM. Markers are represented by black bars.

We conducted whole-genome resequencing of the *F*_1_ population and both parents, generating 232.04 Gb of clean data. The average coverage depths of the genomes were 34-fold for *Vv*MF, 27-fold for *Vd*0940, and 6-fold on average for their offspring. After filtering the single-nucleotide polymorphism (SNP) markers, we constructed separate genetic maps of the two parents and an integrated genetic map using high-quality SNP markers. The female parent *Vv*MF had a genetic map distance of 3229 cM, consisting of 7230 SNP markers distributed over 19 linkage groups (LGs). The total number of SNP markers ranged between 199 and 572 across all linkage groups. We observed the greatest gap, of 55.9 cM, in LG 2, while the smallest was 6.3 cM in LG 8. The percentage of gap < 5 cM (adjacent marker distance < 5 cM) ranged between 97.0% and 99.7% among 19 linkage groups. (Supplementary Data Table S2). For the male parent *Vd*0940, 3040 SNP markers were identified in 19 linkage groups, spanning a distance of 2264 cM. The number of markers detected in each linkage group varied from 79 (LG 3) to 229 (LG 18). The largest gap was 96.6 cM in LG 10, and the percentage of gap <5 cM was between 97.0 and 99.7% in 19 linkage groups (Supplementary Data Table S3). The integrated map comprised 9337 high-quality SNP markers across 19 linkage groups, with a total genetic distance of 3076 cM. The number of SNP markers ranged from 314 to 791 among the various linkage groups. The average genetic distance between markers was 0.3 cM, with a range of 117.38–208.53 cM. The largest gap ranged from 4.1 cM (LG 18) to 18.1 cM (LG 7) in different linkage groups. Notably, the percentage of gap <5.0 cM was 100% for LG 16 and LG 18 (Fig. 3, Supplementary Data Table S4).

### Quantitative trait locus analysis

**Figure 4:**
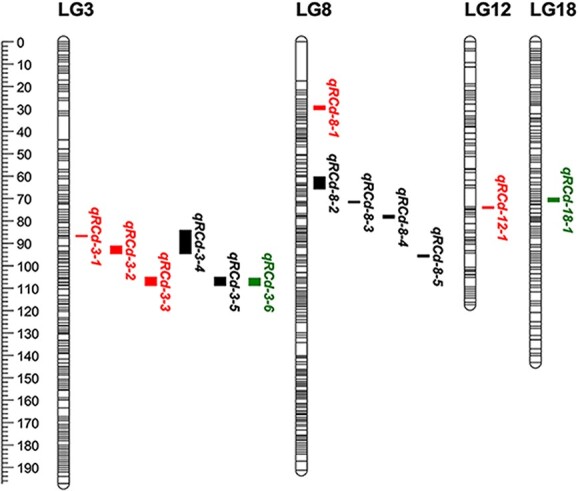
QTLs for *C. diplodiella* resistance in the integrated map of the *Vv*MF × *Vd*0940 population. QTLs are named qRcd-X-Y for 2019 (red), 2020 (black), and 2021 (green), where q represents QTL, Rcd represents resistance to *C. diplodiella*, X represents LG, and Y represents the number of QTLs that appear in this linkage group.

We performed QTL mapping by utilizing the integrated genetic map in conjunction with phenotypic data collected over a 3-year period (2019–21) and mapped them to QTL on Chr 3, 8, 12, and 18 (Fig. 4). Intriguingly, only one stable QTL, *Rcd1* (resistance to *C. diplodiella 1*), was consistently detected on LG 3 over the 3-year period. The maximum LOD value in *Rcd1* was 3.98, explaining up to 17.9% of the phenotypic variation (Table 2). Notably, a stable expression QTL was also detected on LG 3 using two parental maps. By mapping SNP marker positions in the parental QTL intervals to the grape physical map, we observed that the QTL mapping results using the *Vd*0940 map were consistent with the physical positions of the integrated map. The physical location of QTL mapping using the *Vv*MF map is also within the physical location interval of the integrated map (Supplementary Data Table S5). Furthermore, we detected several disease resistance QTLs in 2019–21 using the integrated map, with LOD values ranging from 3.07 to 4.16 and explaining 14.3 and 18.6% of phenotypicvariation, respectively (Supplementary Data Table S6).

**Figure 5:**
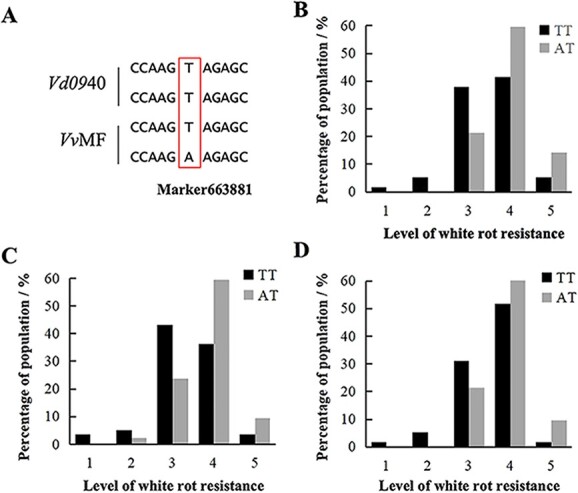
Presence of TT or TA alleles at Marker 663881 distinguished *C. diplodiella* resistance levels among hybrids. **A** Base data of Marker 663881 in parents and the flanking sequence. **B**–**D** Phenotype rating of *F*_1_ progeny with TT and TA in 2019 (**B**), 2020 (**C**), and 2021 (**D**).

In 3 years, 22 markers that co-segregated with *C. diplodiella* resistance were found using a rank sum test based on the Kruskal–Wallis method. Among them, Marker 663881 was found to be most significantly associated with *C. diplodiella* resistance, and was also located in close proximity to the LOD peak in the *Rcd1* region (Table 2, Supplementary Data Table S7). The position of this marker on Chr 3 was 7 653 321 bp. Further analysis of the raw sequencing data linked with this marker revealed that the nucleotides in *Vd*0940 were T/T, whereas the nucleotides in *Vv*MF were T/A. In comparison with T/A individuals with a susceptible phenotype, progeny carrying T/T usually showed a resistant phenotype, and 3-year findings are generally consistent (Fig. 5).

### Quantitative trait locus candidate gene analysis

Based on the grapevine genome sequence, *Rcd1* was located at physical positions of 6 282 673–9 416 779 bp on Chr 3 and contained 185 predicted genes (Supplementary Data S4). Of these predicted genes, 40 were annotated to be associated with disease. To further investigate the potential involvement of these genes in disease resistance, we performed expression gene analysis on grape leaves of *Vv*MF and *Vd*0940 at 24 and 48 h after *C. diplodiella* infection during RNA-seq analysis. Only genes with FPKM (fragments per kilobase of transcript per million mapped reads) values ≥1 were considered expressed genes (Table 3). For these 19 genes, one gene (100246419) had higher expression in *Vd*0940 compared with *Vv*MF at 24 and 48 h after *C. diplodiella* infection, indicating a significant role in grape white rot resistance.

### Functional analysis of *PR1*

**Figure 6:**
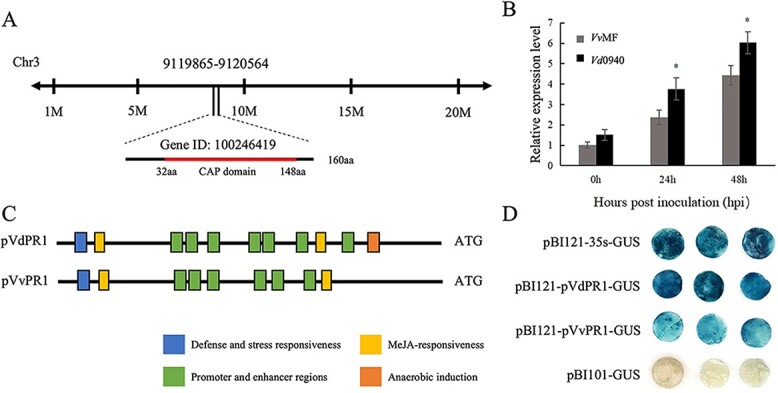
Sequence and expression analysis of PR1 and its promoter. **A** Chromosomal location analysis of *PR1.***B** Leaves of *Vv*MF and *Vd*0940 were infected with *C. diplodiella* and collected for qRT–PCR at 0, 24, and 48 h. *P < .05. **C** Schematic diagram of constructs of the structural difference between the promoters of *VvPR1* (pVvPR1) and *VdPR1* (pVdPR1). **D** Histochemical GUS expression assay. Significance of differences was determined by *t* test.

The pathogenesis-related protein 1 gene (*PR1*, gene ID 100246419) was found on Chr 3 with a full-length coding sequence of 483 bp, encoding 160 amino acids and including a CAP conserved domain, according to the Grape Genome Browser at NCBI (Fig. 6A). To understand the molecular basis for *PR1* expression, we investigated the genomic sequence and promoter activation of the *PR1* gene. The *PR1* genomic sequence analysis indicated that the *VdPR1* (*Vd*0940-PR1) coding region was identical to the *VvPR1* (*Vv*MF-PR1) coding region at the amino acid level (Supplementary Data Fig. S3). However, there was a significant difference in expression patterns at 24 and 48 h after *C. diplodiella* inoculation between *Vd*0940 and *Vv*MF (Fig. 6B). The sequence similarity between the promoters of *VdPR1* and *VvPR1* was calculated to be 96.7%. (Supplementary Data Fig. S4) and contained similar *cis*-acting elements related to the expression of pathogens and stress responses. Nonetheless, some differences were observed in the PlantCARE database analysis (Fig. 6C, Supplementary Data S5). We examined the promoter activities of the *VvPR1* and *VdPR1* genes by histochemical staining to detect β-glucuronidase (GUS) activity in agro-infiltrated tobacco leaves. The GUS-staining blue colors in *VdPR1* were more intense than those in *VvPR1*, indicating that the promoter activity for *VdPR1* is stronger than that for the *VvPR1* genes examined (Fig. 6D).

**Figure 7:**
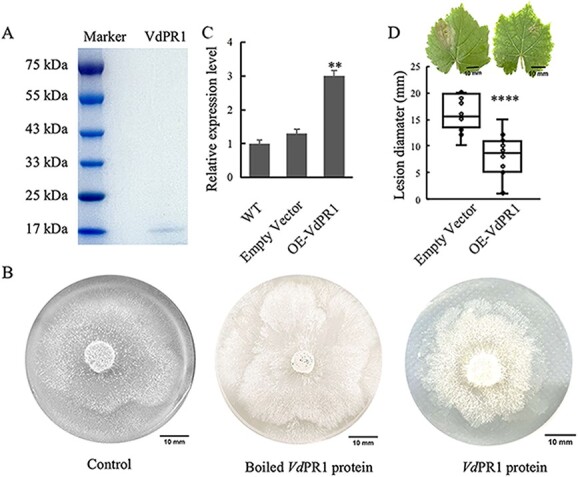
Antimicrobial activity of PR1. **A** Purified recombinant *Vd*PR1 protein. **B** Antifungal activity of PR1 protein (40 μg/ml) against *C. diplodiella* by disk diffusion assay. Protein-free buffer (control) and boiled recombinant VdPR1 proteins were used as negative controls. **C** RT–qPCR analysis of PR1 in infiltrated leaves after 1 day. **P < .01; significant difference between infiltrated leaves [overexpression (OE) empty vector and OE-VdPR1] and WT leaves. **D** Lesion diameter of infiltrated leaves between OE-empty vector and OE-PR1 after *C. diplodiella* inoculation at 26°C. ****P < .0001; significant difference between OE-empty vector and OE-PR1.Significance of differences was determined by *t* test.

Whether PR1 protein plays a role in white rot resistance is not known. We selected the *VdPR1* gene for functional studies because the coding sequences of *Vv*MF and *Vd*0940 were completely consistent. We first purified the PR1 protein and subjected it to an inhibition assay against *C. diplodiella.* Results showed that recombinant *VdPR1* protein incubated at 40 μg/ml for 4 days had a strong inhibitory effect on the growth of *C. diplodiella*, as compared with the no-protein buffer (control) and boiled recombinant *Vd*PR1 protein, which had no effect on *C. diplodiella* growth (Fig. 7A and B). To further validate the role of *Vd*PR1 in enhancing resistance against *C. diplodiella*, we transiently transformed the *PR1* gene into disease-susceptible grape (Jingxiu) leaves and inoculated them with agar disks containing mycelium of white rot. Transcript levels of *PR1* were analyzed at 24 h after infiltration, and results showed that the overexpressed *PR1* gene had a 3-fold higher expression level compared with untransformed leaves and empty vector, confirming successful overexpression of *PR1* (Fig. 7C). Lesion diameters were then measured 3 days after pathogen inoculation, and we found that overexpressed *VdPR1* significantly reduced lesion diameter compared with the overexpressed empty vector, indicating that transient *VdPR1* overexpression enhances *C. diplodiella* resistance in disease-susceptible grape varieties (Fig. 7D).

## Discussion

In grapevine, genetic maps are typically generated by crossing two heterozygous parents and evaluating their segregating markers in the offspring [54]. The genetic maps based on first-generation and second-generation markers, such as RFLP, AFLP, RAPD, and SSR, have resulted in low map marker density, large marker spacing across linkage groups, and uneven marker distribution throughout the genome [11]. With the advancement of sequencing technology, high-density genetic maps using SNP markers have greatly increased the level of fine localization [54–56]. Constructed genetic maps using the PCR method had a density range of 3.9–12.7 cM, whereas those using the SNP method had a density range of 0.41–1.81 cM [55, 57–59]. Although the genetic distance between markers is decreasing, the populations used to construct the genetic map had an average of 166 offspring individuals [54]. Here, we constructed a high-density linkage genetic map with an average marker interval of 0.3 cM using two parents (*Vd*0940 and *Vv*MF) and a mapping population of 101 individuals. Additionally, the average Spearman correlation coefficient of positions in genetic and physical maps was 0.94 (Supplementary Data Fig. S5). The high collinearity indicates that the markers accurately cover the 19 chromosomes and sufficiently represent the *Vitis* genome. Therefore, our genetic map provides valuable information for QTL analysis, gene mapping, and marker-assisted selection to enhance grape resistance to white rot.

In resistance studies, the accuracy of resistance assessment and phenotypic data is crucial. The evaluation of grapevine resistance under *in vitro* conditions enables an increased degree of bioassay replication and allows for the execution of one or more experiments per year, rendering the *in vitro* phenotyping strategy an efficient and practical tool for identifying resistance QTLs [60]. Phenotypic analyses in resistance mapping studies usually use visual ratings of disease symptoms. Particularly in the study of resistance loci for downy mildew (DM) and powdery mildew (PM), DM and PM sporulation is evaluated for incidence and severity using different visual scales, such as the OIV452-1 and 455-1 descriptors [60, 61]. In the pathogenicity evaluation of white rot fungal strains, Chethana *et al*. [7] recorded lesion length 3 days after inoculation to determine that the most pathogenic strain was JZB3700012. Although visual evaluation is considered subjective, the impact of human influence can be mitigated by employing biological replication and multi-year testing [60]. In this study, we ensured the accuracy of the phenotypes by collecting four leaves from each plant and inoculating four spots on each leaf for 3 years. Importantly, there was a significant correlation between the phenotypic data in each year.

Identifying QTLs and candidate genes associated with white rot resistance in grapes is of utmost importance for the industry. In this study, we confirmed one stable QTL for white rot resistance on Chr 3, explaining up to 17.9% of the phenotypic variation. Only one report on QTL for grapevine white rot resistance has been published so far. Su *et al*. [25] identified one stable QTL for white rot resistance locus on chromosome 14 through the 'Zhuosexiang' × 'Victoria' population, explaining 13.43% of the phenotypic variation. Various localization results are frequently found utilizing different populations for the same disease. Notably, we found that individuals carrying the homozygous T/T genotype were usually resistant to white rot. Similarly, Su *et al*. [25] found that individuals carrying the homozygous G/G (Chr14_3929380) genotype had smaller lesion areas and were resistant to *C. diplodiella*, while those carrying the heterozygous G/A genotype showed susceptibility. In QTL mapping of DM resistance, Bhattarai *et al*. [12] found that individuals carrying the homozygous genotype showed higher resistance to DM. Additionally, a marker closely associated with sugarcane yellow leaf virus (SCYLV) resistance was found in sugarcane, with homozygous (C/C) progeny effectively suppressing the incidence of SCYLV [62]. Several disease-resistance-related genes, including disease-resistance protein (NBS-LRR class), protein trichome birefringence, pathogenesis-related protein, zinc finger protein, E3 ubiquitin-protein and serine/threonine-protein kinase, were found in the QTL region. Some of the 386 *R* genes predicted for the grapevine are related to resistance to powdery mildew, white rot, downy mildew, and anthracnose [63–66]. Numerous NBS-LRR-like *R* genes were also identified in previously identified disease resistance QTL regions. For example, 11 NBS-LRR genes were found in the comparable region of *Cgr1* [57]. Two of the seven TIR-NB-LRR genes in the relevant region of the *Rpv1*/*Run1* locus have been demonstrated to increase disease resistance using transgenic verification [67]. Additionally, an NBS-LRR-like *R* gene that may be crucial in grape white rot resistance was discovered [25]. Upregulation of *PR1* expression is often used as a marker of resistance responses, such as PTI and SAR [68]. The *PR1* gene was found to be important for grape resistance to white rot [33].

In this study, we also identified QTLs located in Chr 8, Chr 12, and Chr 18 with lower reproducibility. This may be attributed to the fact that QTLs with substantial effects can be identified in small populations, whereas QTLs with weaker effects require larger population sizes to be detected [69]. We found these loci present in multiple disease-resistance genes. The receptor-like kinases on the cell membrane serve as PRRs that are crucial for the detection of PAMPs, a process commonly known as PTI [26]. Wang has reported that the lectin receptor-like kinases (LecRKs) can enhance resistance to powdery mildew in wheat [70]. Pathogens can counteract PTI by secreting effectors, but R proteins in plants can recognize these effectors and trigger an immune response (ETI), leading to programmed cell death, inhibiting pathogen spread [28, 29]. Both PTI and ETI can cause downstream responses such as reactive oxygen species (ROS) burst, MAPK cascade activation, and PR protein induction [28, 71]. These intricate defense responses involve the expression of numerous defense genes. The expression of defense-related genes is regulated by transcription factors (TFs), which work in concert with other proteins to bind to the DNA-binding sites of target genes [72]. Our results indicate that the expression level of the receptor-like kinase gene (gene ID 100854743, Chr 8; 109121483, Chr 12), peroxidase (gene ID 100854817, Chr 12), *WRKY49* (gene ID100254510, Chr 8), E3 ubiquitin-protein ligase (gene ID 100257429, Chr 12) was higher in *Vd*0940 than *Vv*MF induced by *C. diplodiella* after 24 and 48 h.

Based on the results of QTL mapping and RNA-seq analysis, we hypothesized that the *PR1* gene plays a significant role in white rot resistance. Even though the coding sequences of *PR1* genes in *Vd*0940 and *Vv*MF are identical, their expression patterns in response to *C. diplodiella* infection were significantly different. During the infection phase, the level of induced expression of *VdPR1* was significantly higher than that of *VvPR1*, which may be a contributing factor to the much higher resistance of *Vd*0940 to *C. diplodiella* (Fig. 6B). Previous studies have demonstrated that the *cis*-acting sequence in the plant promoter is a critical element in controlling plant gene expression, and GUS reporter gene-aided histochemistry can be utilized to study gene expression at the transcriptional level [73, 74]. Thus, the stronger promoter activity for *VdPR1* compared with *VvPR1* was the primary reason for the difference in gene expression (Fig. 6D). Our findings indicated that the PR1 protein can inhibit the growth of *C. diplodiella* (Fig. 7B). Recent research has shown that the *in vitro* activity of PR1 orthologs from various plant species can inhibit the growth of many pathogens [75–77]. Furthermore, we observed that overexpression of *PR1* in susceptible *V. vinifera* enhanced plant resistance to the pathogen (Fig. 7D). Similarly, overexpression of *PR1* in tobacco improved resistance to *Botrytis cinerea*, while overexpression of *PR1* in transgenic *Arabidopsis* exhibited higher resistance to *Sclerotinia sclerotiorum* [78, 79]. Additionally, the effector protein of the pathogen can be targeted by PR1. For example, Wheat PR1 proteins were shown to interact with the effector proteins of *Stagonospora nodorum* (SnToxA and SnTox3), barley powdery mildew effector (CSEPP0055) targeted PR1a and PR1b, and *S. sclerotiorum* effector (SsCP1) interacted with PR1. These findings suggested that PR1 is a common host protein implicated in host defense against pathogens [79–83]. Therefore, further studies are required to determine whether *C. diplodiella* effector proteins can target PR1.

## Materials and methods

### Plant materials

The *F*_1_ population was generated by crossing ‘Manicure Finger’ (*V. vinifera*, female, *Vv*MF) with ‘0940’ (*V. davidii*, male, *Vd*0940) in 2015. The crosses were manually performed in the field by removing the floral caps on the *Vv*MF parent and applying dried pollen collected from the *Vd*0940 parent. To prevent unintended pollination, inflorescences were covered with paper bags for 4 weeks. Subsequently, seeds were collected from berries in October of the same year. The germination of seeds took place in the greenhouse during the spring of 2016, and the resulting seedlings were transferred to the National Grape Germplasm Resource Garden (Zhengzhou) for field management. The seedlings were cultivated in accordance with the appropriate field management procedures during their early growth stages. Eventually, 101 individual plants survived and were used for the mapping population. Nevertheless, the *F*_1_ populations were not treated with fungicides in the current-year trials to evaluate their resistance to white rot.

### Disease evaluation

The *C. diplodiella* (Speg.) Sacc. strain WR01 was obtained from the Institute of Plant Protection, Chinese Academy of Agricultural Sciences. The *C. diplodiella* was grown in the dark at 28°C on potato dextrose agar (PDA) medium until the mycelium covered the whole medium. Mature leaves were collected from each individual from the vineyard. Leaf surface sterilization was performed as described by Chethana et al. [7], [10]. Wound inoculation experiments were conducted using five leaves per plant (including one control) and each leaf was wounded on the upper epidermal layer and divided into four parts on average. PDA blocks (9 mm in diameter) containing fungal mycelium were placed on the wounds while sterile agar blocks (9 mm in diameter) were placed on the punctured leaves in the control treatment. The leaves were placed on moist filter paper within Petri dishes and incubated at 28°C. Three days after inoculation, we measured the average lesion diameter of necrotic patches using a slide caliper rule and rated disease severity on a scale of 1–5 (Table 1). In each experiment, both parental leaves were inoculated as resistant and susceptible controls. We conducted the experiment in July of 2019, 2020, and 2021 and averaged phenotypic data for lesion diameter from four biological replicates per individual (Supplementary Data S1).

**Table 1:** Rating scale for characterization of white rot infestation in experiments.

Rating	Average lesion diameter (mm)
1	≤10
2	10–20
3	20–30
4	30–40
5	>40

**Table 2:** Description of stable QTLs detected for *C. diplodiella* resistance in the integrated maps of *Vv*MF × *Vd*0940 throughout 3 years.

Year	LG	LOD threshold^a^	Peak LOD	Confidence interval (cM)	Peak position (cM)	Co-segregated marker	PVE (%)
2019	3	3.1	3.85	105.077–108.803	107.164	Marker 663881	17.4
2020	3	3.1	3.98	105.077–108.803	107.164	Marker 663881	17.9
2021	3	2.9	3.75	105.596–108.803	107.164	Marker 663881	17

a Calculated threshold values using a permutation test at α = 0.05.

**Table 3:** Candidate genes associated with resistance to grape white rot.

Gene ID	FPKM						Annotation
	*Vv*MF			*Vd*0940			
	0 h	24 h	48 h	0 h	24 h	48 h	
100250734	1.4	1.6	1.1	1.3	2.2	1.6	Pentatricopeptide repeat-containing protein
100855373	2.1	2.9	1.6	0.8	2.6	2.0	Putative disease resistance protein (NBS-LRR)
100256695	0.3	0.1	0.1	3.5	2.2	2.4	Serine carboxypeptidase-like 13
100264703	0.3	2.8	1.8	0.2	5.2	2.7	Zinc finger protein
100257649	0.3	1.9	1.0	1.2	5.0	2.9	Pentatricopeptide repeat-containing protein
100256594	1.6	4.4	4.6	2.6	5.5	5.0	Protein trichome birefringence-like 13
100265141	0.0	0.0	6.6	0.0	0.0	5.3	Basic form of pathogenesis-related protein 1-like
100247990	15.0	18.9	151.2	1.0	1.5	8.5	Basic form of pathogenesis-related protein 1
100262780	8.6	17.1	16.1	16.8	13.1	12.3	Thioredoxin domain-containing protein 9 homolog
100259392	1.4	3.8	3.2	3.8	17.1	13.5	Zinc finger SWIM domain-containing protein 7
100249713	9.7	25.2	15.7	9.6	20.6	16.0	Serine/threonine-protein kinase TOR
100242874	6.7	13.8	13.6	17.0	18.5	16.8	Zinc finger protein-like 1
100242852	10.8	5.1	483.2	0.2	1.6	22.7	Pathogenesis-related leaf protein 6
100265220	2.8	48.1	5.1	1.2	16.2	25.8	Serine carboxypeptidase-like 16
100249808	183.5	40.6	38.5	123.1	12.5	26.6	Probable E3 ubiquitin-protein ligase
100254953	153.2	21.8	54.0	92.8	13.7	48.4	Probable protein phosphatase 2C 63
100 46419	6.4	16.4	34.1	1.7	17.5	51.4	Pathogenesis-related protein 1
100258414	136.7	187.0	920.0	22.9	179.4	521.8	Pathogenesis-related protein
100258264	1098.0	1305.9	894.0	779.6	34.5	1194.6	Zinc finger protein ZAT10

### DNA extraction and library building

Genomic DNA was extracted from both the parent and progeny leaves using a DP360 genomic DNA extraction kit (Tiangen Biotech, China). The purity of the DNA was determined through agarose gel analysis. Next, 200- to 500-bp fragments were randomly cut using sonication, and 3′A and sequencing adapters were added. The resulting samples were purified and amplified via PCR to develop the library. Subsequently, the HiSeq2500 system (Illumina, San Diego, CA, USA) was utilized to re-sequence the entire genome. The average coverage depths of the genomes were 34-fold for *Vv*MF, 27-fold for *Vd*0940, and 6-fold on average for their offspring (Supplementary Data S2).

### Genotyping and genetic map construction

A 12× assembly of the grape PN40024 genome was used as a reference [34]. Prior to alignment, the reads underwent Fastp [35] processing to eliminate adapters, N >10% of read pairs, and low-quality bases (Q <10) >50% of read pairs. Using the Burrows–Wheeler Aligner (BWA) tool, the reference genome was aligned with clean reads. SNP marker detection and filtering were performed using GATK [36]. The SNP markers were discovered based on parental sequence depths exceeding 6-fold and offspring sequence integrity >75%. Additionally, map development initially excluded markers with significant segregation distortion (*P* < .01). High-quality SNP markers were partitioned into 19 groups. After determining the modified logarithms of odds (MLOD) values of markers for each linkage group, markers with MLOD values >3 were ranked. A LOD value of >3.0 implies that the chances are greater than 1000:1 that the markers are linked for a given recombination estimate [37]. Finally, we utilized HighMap [38] software to evaluate the arrangement of markers in each linkage group and estimate the genetic distance between nearby markers, resulting in the final genetic map (Supplementary Data S3).

### Quantitative trait locus analysis and candidate gene prediction

To detect QTLs, we employed the MapQTL6.0 [39] software on the integrated map, combining it with the phenotypic data used for each year alone. We repeated this analysis with separate parental maps for comparison. The initial step involved using interval mapping (IM) to detect potential QTLs, and a significant QTL was defined based on LOD >3.0 [40, 41]. After identifying a potential QTL using interval mapping analysis, we utilized markers that linked to the QTL as cofactors. For more precise positioning of QTLs, we employed multiple QTL model (MQM) mapping with selected cofactors [41, 42]. To determine the LOD threshold required for a QTL to be present in a specific genomic region, we conducted a permutation test with 1000 cycles. We considered a locus to be present if its confidence interval was higher than the LOD threshold. Finally, all SNP markers located in the QTL intervals of the integrated map were utilized to identify candidate genes by locating their physical position on the grape PN40024 genome [43, 44]. MapChart 2.2 was used to show the QTL regions of the linkage map [45].

### Transcriptome analysis and qRT–PCR validation

The collected leaves were inoculated as described previously. We chose infected grape leaves 24 and 48 h post-infection of *Vv*MF and *Vd*0940, and non-infected leaves (0 h) as a control, using three replicates per cultivar (Supplementary Data Fig. S2). Total RNA was extracted from entire leaves of *Vv*MF and *Vd*0940 using the Plant Total RNA Isolation Kit (DP441, Tiangen Biotech, China) in accordance with the manufacturer’s instructions. Subsequently, cDNA was synthesized using the PrimeScript™ RT Reagent Kit (KR116, Tiangen Biotech, China). RNA sequencing was performed using the DNBSEQ platform (MGI Tech, China). High-quality clean reads were obtained by filtering raw reads using SOAPnuke. The clean reads were then compared with the reference gene sequence using Bowtie2. Gene expression levels were calculated using RNA-Seq by Expectation Maximization (RSEM) [46] and were expressed as FPKM. The datasets can be found at NCBI under BioSample accession PRJNA938012. To evaluate the transcription levels of *Vv*MF and *Vd*0940 candidate genes, qPCR primers (qRTPCR-PR1, *Vv*Actin) were designed using Primer3plus (https://www.primer3plus.com/). The expression level was determined as 2^−ΔΔCt^, normalized to the Ct value of *Vv*Actin [47]. Details of the primers utilized are provided in Supplementary Data Table S1.

### Cloning and analysis of gene sequences

The *PR1* reference sequences were downloaded from the GenBank database of the NCBI (https://www.ncbi.nlm.nih.gov/gene/100246419). To clone the *PR1* coding sequence, primers (PR1CDS) were designed using the *PR1* coding sequence from the grapevine database. Using SignalP6.0 (https://services.healthtech.dtu.dk/service.php?SignalP-6.0), signal peptides were predicted. The promoter sequence of PR1primer (PR1 promoter) was designed based on the upstream sequences 2000 bp of *PR1* in the grapevine database. The promoter sequence was analyzed with the PlantCARE database [48]. Aligning multiple sequences was performed by DNAMAN (Lynnon Biosoft; San Diego, CA, USA). The primers used are provided in Supplementary Data Table S1.

### Construction of β-glucuronidase vectors and histochemical β-glucuronidase staining

The GUS reporter gene was fused to the *PR1* promoter using primer (pBI121-*Vv*PR1promoter and pBI121-*Vd*PR1promoter) in the expression vector pBI-121. pBI-121, an expression vector containing the CaMV35 strong promoter, and pBI-101, a non-promoter expression vector, served as positive and negative controls, respectively. Transient expression assay using *Agrobacterium* was as described by Rahman *et al*. [49]. The *Agrobacterium* suspension was infiltrated into the tobacco leaf, which was placed in a dark room at 26°C for 24 hours after infiltration, then moved to a growth chamber. Histochemical staining was used for the measurement of β-glucuronidase (GUS) expression for qualitative analysis of promoter activity [50]. Following the manufacturer’s protocol, agro-infiltrated tobacco leaves were stained with the Gusblue kit (GT0931, Huayueyang Biotech, China) and chlorophyll was removed from tobacco leaves by using ethanol. The primers used are provided in Supplementary Data Table S1.

### Purification of recombinant PR1 protein

In order to express the PR1 protein with the His tag protein, the coding region without the signal peptide sequence was amplified using primer (PR1-no signal peptide) and cloned into the pEASY-Blunt1 (CE111-01, TransGen Biotech, China) vector, which was then introduced into BL21.After incubating transformed BL21 bacteria in Luria–Bertani (LB) broth at 37°C to an optical density of OD_600_ = 0.6, a final dosage of 0.6 mM of isopropyl d-1-thiogalactopyranoside (IPTG) was added to promote PR1 production. At 12 hours after induction at 19°C, bacterial cells were extracted by centrifugation and suspended in a balanced buffer (300 mM NaCl, 30 mM NaH_2_PO_4_, and 10 mM imidazole, pH 8) and lysed with an ultrasonic cell crusher. The manufacturer’s instructions were followed to purify His6-tagged proteins from bacterial extracts using Ni-NTA resin (DP101-01, TransGen Biotech, China). Using BSA as a standard, the Lowry method was used to measure protein concentration. The used primers are provided in Supplementary Data Table S1.

### Antifungal activity determination *in vitro*

The *in vitro* antifungal activities of PR1 protein were investigated by disk diffusion assays with modifications described by Zandvakili *et al*. [51]. Recombinant PR1 protein (40 μg/ml) was applied to the solid PDA medium’s surface. Boiled recombinant PR1 protein and protein-free buffer were incubated as control plates. After 4 days of incubation at 28°C without light, the mycelial growth of each sample was assessed.

### Analysis of transient expression of *PR1* in grapevine leaf tissue

The *PR1* coding sequence was cloned with the GFP reporter gene into the plant expression vector pCAMBIA1302 using primer PBI1302-VdPR1 and then injected into GV3101. Cultures were kept at 28°C and 180 rpm for 12 hours in a liquid medium made of lysogenic broth. After 20 minutes of centrifuging at 7000 g, the bacteria that had settled out were resuspended in infiltration buffer (10 mM MES, pH 5.6, 10 mM MgCl_2_, 100 μM acetosyringone) until the OD_600_ reached 0.6. For agro-infiltration, we used leaves from *in vitro*-grown ‘Jingxiu’ plants with similar ages and sizes. The agro-infiltration was carried out as specified by Guan *et al*. [52]. One day after agro-infiltration, the expression of *PR1* was examined in the infiltrated leaves. After 1 day of agro-infiltration, leaves were infected with *C. diplodiella*, as reported by Santos-Rosa *et al*. [53]. The diameter of the lesions was determined 3 days after pathogen inoculation. Three independent biological repeats were performed and each repeat contained four grape leaves. The primers used are provided in Supplementary Data Table S1.

## Supplementary Material

Web_Material_uhad063

## Data Availability

All the data can be found in the main text or the supplements.
