# Supplementary material for: QTL detection and candidate gene analysis of grape white rot resistance by interspecific grape (Vitis vinifera L. × Vitis davidii Foex.) crossing
Source: Hortic Res. 2023 Apr 2;10(5):uhad063. doi: 10.1093/hr/uhad063 (PMC10208900; doi:10.1093/hr/uhad063)
Supplement: Web_Material_uhad063 [file web_material_uhad063.zip › Supplementary20230301 .docx]

Supplementary Table S1 Primers used in this article

| Primer name | Forward Primer | Reverse Primer |
| --- | --- | --- |
| Vvactin | TTACCGCGGGCAAGAGATAC | ATTCACACGCTCCTTTTGGG |
| PR1CDS | ATGGGGTTGTTTAAGATTTCACTAGTTTTTATTTGTCT | TCAATAAGGACGCTGTCCGACA |
| PR1promoter | CATAAAATAGTGTATTTTTTTTTCCCCATTTTCCTCTTTTTCT | TTTCAGTTGTGAAGTTTAATGTAATTGATGTGGAATG |
| pBI121-VvPR1promoter | GACCATGATTACGCCAAGCTTCATAAGATAGTGTATTTTTTTTTTTTTTCATTTTCC | GGACTGACCACCCGGGGATCCTTTCAGTTGTGAAGTTTAATGTAATTGATG |
| pBI121-VdPR1promoter | GACCATGATTACGCCAAGCTTCACAAAATAATGTAATTTTTTTTTTCATTTTCCT | GGACTGACCACCCGGGGATCCTTTCAGTTGTGAAGTTTAATGTAATTGATG |
| qRTPCR-PR1 | TGGAACAACACCGTAGCCTC | CCAGGCAATGTTCTCCCCAT |
| PR1-no signal peptide | GCTCAAAACTCACAGCAGGACT | TCAATAAGGACGCTGTCCGACA |
| PBI1302-VdPR1 | ACGGGGGACTCTTGACCATGGATGGGGTTGTTTAAGATTTCACTAGTTTTTATTTGTCT | AAGTTCTTCTCCTTTACTAGTATAAGGACGCTGTCCGACA |

Supplementary TableS2 Main characteristics of linkage groups in female parent 'Manicure Finger’

| Linkage groups | Map of female parent ‘Manicure Finger’ | | | | | | | |  |
| --- | --- | --- | --- | --- | --- | --- | --- | --- | --- |
|  | Genetic distance (cM) | SNP markers | Average  distance (cM) | | | Max Gap (cM) | | Percentage of Gap < 5 (cM) | |
| 1 | 157.3 | 373 | | 0.4 | 26.9 | | 99.2 | |  |
| 2 | 212.7 | 376 | | 0.6 | 55.9 | | 97.9 | |  |
| 3 | 215.7 | 400 | | 0.5 | 17.6 | | 99.8 | |  |
| 4 | 155.6 | 466 | | 0.3 | 7.5 | | 98.7 | |  |
| 5 | 211.0 | 491 | | 0.4 | 9.8 | | 98.8 | |  |
| 6 | 130.1 | 307 | | 0.4 | 9.8 | | 98.0 | |  |
| 7 | 204.4 | 461 | | 0.4 | 26.9 | | 98.9 | |  |
| 8 | 243.8 | 572 | | 0.4 | 6.3 | | 98.6 | |  |
| 9 | 147.3 | 371 | | 0.4 | 8.6 | | 98.7 | |  |
| 10 | 126.2 | 305 | | 0.4 | 7.5 | | 97.0 | |  |
| 11 | 170.8 | 339 | | 0.5 | 28.6 | | 98.2 | |  |
| 12 | 107.4 | 299 | | 0.4 | 19.1 | | 98.0 | |  |
| 13 | 166.8 | 388 | | 0.4 | 22.0 | | 97.7 | |  |
| 14 | 217.6 | 572 | | 0.4 | 8.6 | | 99.0 | |  |
| 15 | 89.2 | 273 | | 0.3 | 9.8 | | 99.3 | |  |
| 16 | 152.0 | 355 | | 0.4 | 14.9 | | 99.7 | |  |
| 17 | 174.7 | 263 | | 0.7 | 36.2 | | 97.7 | |  |
| 18 | 146.9 | 199 | | 0.7 | 53.0 | | 98.0 | |  |
| 19 | 200.0 | 420 | | 0.5 | 9.8 | | 97.6 | |  |
| Total | 3229 | 7230 | | 0.4 | / | | / | |  |

Supplementary TableS3 Main characteristics of linkage groups in female parent ‘940’

| Linkage groups | Map of female parent ‘940’ | | | | | | | |  |
| --- | --- | --- | --- | --- | --- | --- | --- | --- | --- |
|  | Genetic distance (cM) | SNP markers | Average  distance (cM) | | | Max Gap (cM) | | Percentage of Gap < 5 (cM) | |
| 1 | 93.7 | 165 | | 0.6 | 22.0 | | 97.6 | |  |
| 2 | 85.0 | 166 | | 0.5 | 8.6 | | 97.0 | |  |
| 3 | 141.6 | 79 | | 1.8 | 36.2 | | 87.2 | |  |
| 4 | 150.4 | 179 | | 0.8 | 45.1 | | 96.1 | |  |
| 5 | 68.2 | 113 | | 0.6 | 14.9 | | 96.4 | |  |
| 6 | 89.9 | 101 | | 0.9 | 40.4 | | 96.0 | |  |
| 7 | 177.9 | 163 | | 1.1 | 62.4 | | 97.5 | |  |
| 8 | 111.2 | 148 | | 0.8 | 28.6 | | 98.0 | |  |
| 9 | 91.5 | 162 | | 0.6 | 14.9 | | 98.1 | |  |
| 10 | 151.2 | 114 | | 1.3 | 96.6 | | 96.5 | |  |
| 11 | 112.8 | 141 | | 0.8 | 23.6 | | 97.1 | |  |
| 12 | 89.8 | 171 | | 0.5 | 13.6 | | 95.9 | |  |
| 13 | 182.3 | 150 | | 1.2 | 34.2 | | 92.6 | |  |
| 14 | 164.4 | 227 | | 0.7 | 69.8 | | 98.2 | |  |
| 15 | 115.0 | 160 | | 0.7 | 19.1 | | 95.0 | |  |
| 16 | 68.3 | 184 | | 0.4 | 7.5 | | 98.9 | |  |
| 17 | 114.4 | 178 | | 0.6 | 7.5 | | 98.3 | |  |
| 18 | 123.1 | 229 | | 0.5 | 13.6 | | 98.7 | |  |
| 19 | 133.5 | 210 | | 0.6 | 40.4 | | 98.1 | |  |
| Total | 2264 | 3040 | | 0.7 | / | | / | |  |

Supplementary TableS4 Main characteristics of linkage groups in integrated map

| Linkage groups | Integrated map | | | | | | |  |
| --- | --- | --- | --- | --- | --- | --- | --- | --- |
|  | Genetic distance (cM) | SNP markers | Average  distance (cM) | | Max Gap (cM) | | Percentage of Gap < 5 (cM) | |
| 1 | 139.7 | 534 | | 0.3 | 10.3 | 99.6 | |  |
| 2 | 162.1 | 314 | | 0.5 | 11.8 | 99.1 | |  |
| 3 | 197.2 | 463 | | 0.4 | 10.8 | 99.8 | |  |
| 4 | 164.1 | 637 | | 0.3 | 15.9 | 99.5 | |  |
| 5 | 194.6 | 340 | | 0.6 | 8.1 | 99.2 | |  |
| 6 | 129.1 | 406 | | 0.3 | 9.8 | 98.8 | |  |
| 7 | 197.1 | 621 | | 0.3 | 18.1 | 99.4 | |  |
| 8 | 191.3 | 714 | | 0.3 | 17.5 | 99.7 | |  |
| 9 | 141.8 | 529 | | 0.3 | 6.3 | 99.2 | |  |
| 10 | 151.2 | 418 | | 0.4 | 17.2 | 98.3 | |  |
| 11 | 170.8 | 479 | | 0.4 | 17.3 | 98.5 | |  |
| 12 | 117.4 | 465 | | 0.3 | 8.4 | 98.9 | |  |
| 13 | 179.4 | 534 | | 0.3 | 17.2 | 98.3 | |  |
| 14 | 208.5 | 791 | | 0.3 | 6.8 | 99.6 | |  |
| 15 | 130.2 | 428 | | 0.3 | 13.6 | 99.1 | |  |
| 16 | 119.6 | 351 | | 0.3 | 4.3 | 100.0 | |  |
| 17 | 149.2 | 438 | | 0.3 | 5.2 | 99.8 | |  |
| 18 | 143.2 | 423 | | 0.3 | 4.1 | 100.0 | |  |
| 19 | 189.4 | 452 | | 0.4 | 14.8 | 98.7 | |  |
| Total | 3076 | 9337 | | 0.3 | / | / | |  |

Supplementary TableS5 The QTL for *C. diplodiella* resistance using both parents map

| MAP | Year | Chr | LOD threshold^a^ | Peak Lod | Confidence interval | Marker  interval | PVE(%) | Physical  position |
| --- | --- | --- | --- | --- | --- | --- | --- | --- |
| *Vd*0940 | 2019 | 3 | 2.8 | 3.85 | 65.73-67.73 | Marker638599-Marker678198 | 17.4 | 4954300-9416779 |
|  | 2020 | 3 | 2.7 | 4.17 | 58.27-67.73 | marker635032-Marker678198 | 18.6 | 4535710-9416779 |
|  | 2021 | 3 | 2.8 | 3.75 | 66.73-67.73 | Marker651840-Marker678198 | 17 | 6282673-9416779 |
|  |  |  |  |  |  |  |  |  |
| *Vv*MF | 2019 | 3 | 2.8 | 3.48 | 88.308-92.329 | Marker646495-Marker660592 | 15.8 | 5779916-7287503 |
|  |  | 3 | 2.8 | 3.85 | 116.597-118.597 | Marker662747-Maker667716 | 17.4 | 7525855-8114309 |
|  | 2020 | 3 | 2.8 | 3.69 | 85.246-92.329 | Marker644306-Marker660592 | 16.7 | 5572025-7287503 |
|  |  | 3 | 2.8 | 3.98 | 116.597-118.597 | Marker662747-Maker667716 | 17.9 | 7525855-8114309 |
|  | 2021 | 3 | 2.5 | 3.75 | 116.597-118.597 | Marker662747-Maker667716 | 17 | 7525855-8114309 |

^a^ Calculated threshold values using a permutation test at α = 0.05

Supplementary TableS6 Summary of the QTL for *C. diplodiella* resistance using integrated map

| Year | LG | LOD threshold^a^ | Confidence interval (cM) | Peak Position(cM) | Peak LOD | PVE (%) |  |
| --- | --- | --- | --- | --- | --- | --- | --- |
|  |  |  |  |  |  |  |  |
| 2019 | 3 | 3.1 | 86.493-86.993 | 86.493 | 3.2 | 14.7 |  |
|  | 3 |  | 91.205-94.568 | 93.205 | 3.49 | 15.9 |  |
|  | 3 |  | 105.077-108.803 | 107.164 | 3.85 | 17.4 |  |
|  | 8 |  | 28.603-30.283 | 28.603 | 3.12 | 14.3 |  |
|  | 12 |  | 73.552-74.444 | 73.684 | 3.3 | 15.1 |  |
|  |  |  |  |  |  |  |  |
| 2020 | 3 | 3.1 | 84.173-94.568 | 87.653 | 4.16 | 18.6 |  |
|  | 3 |  | 105.077-108.803 | 107.164 | 3.98 | 17.9 |  |
|  | 8 |  | 60.295-65.682 | 62.95 | 3.51 | 16 |  |
|  | 8 |  | 71.159-71.935 | 71.159 | 3.81 | 17.2 |  |
|  | 8 |  | 77.418-78.777 | 77.418 | 3.58 | 16.2 |  |
|  | 8 |  | 95.05-96.066 | 96.066 | 3.39 | 15.5 |  |
|  |  |  |  |  |  |  |  |
| 2021 | 3 | 2.9 | 105.596-108.803 | 107.164 | 3.75 | 17 |  |
|  | 18 |  | 69.775-71.43 | 71.43 | 3.7 | 16.1 |  |

^a^ Calculated threshold values using a permutation test at α = 0.05

Supplementary Table S7 Identification of markers co-segregated with resistance to C.diplodiella using the Kruskal–Wallis algorithm

| Locus | LG | Df^a^ | Significance level | | | |
| --- | --- | --- | --- | --- | --- | --- |
|  |  |  | 2019 | | 2020 | 2021 |
| Marker655251 | 3 | 1 | *** | | **** | ** |
| Marker656426 | 3 | 1 | *** | | **** | ** |
| Marker663881 | 3 | 1 | **** | | **** | ** |
| Marker651840 | 3 | 1 | ** | | ** | ** |
| Marker651917 | 3 | 1 | ** | | ** | ** |
| Marker653959 | 3 | 1 | ** | | ** | ** |
| Marker661751 | 3 | 1 | ** | | ** | ** |
| Marker662332 | 3 | 1 | ** | | ** | ** |
| Marker664145 | 3 | 1 | ** | | ** | ** |
| Marker664326 | 3 | 1 | ** | | ** | ** |
| Marker665083 | 3 | 1 | ** | | ** | ** |
| Marker666805 | 3 | 1 | ** | | ** | ** |
| Marker667643 | 3 | 1 | ** | | ** | ** |
| Marker668176 | 3 | 1 | ** | | ** | ** |
| Marker668230 | 3 | 1 | ** | | ** | ** |
| Marker670111 | 3 | 1 | ** | | ** | ** |
| Marker670940 | 3 | 1 | ** | | ** | ** |
| Marker667716 | 3 | 1 | *** | | *** | ** |
| Marker672442 | 3 | 1 | ** | | ** | ** |
| Marker673704 | 3 | 1 | ** | | ** | ** |
| Marker676005 | 3 | 1 | ** | | ** | ** |
| Marker678198 | 3 | 1 | ** | | ** | ** |
| *P = **0.01, ***0.001, ****0.0001. ^a^Degrees of freedom | | | |  |  |  |


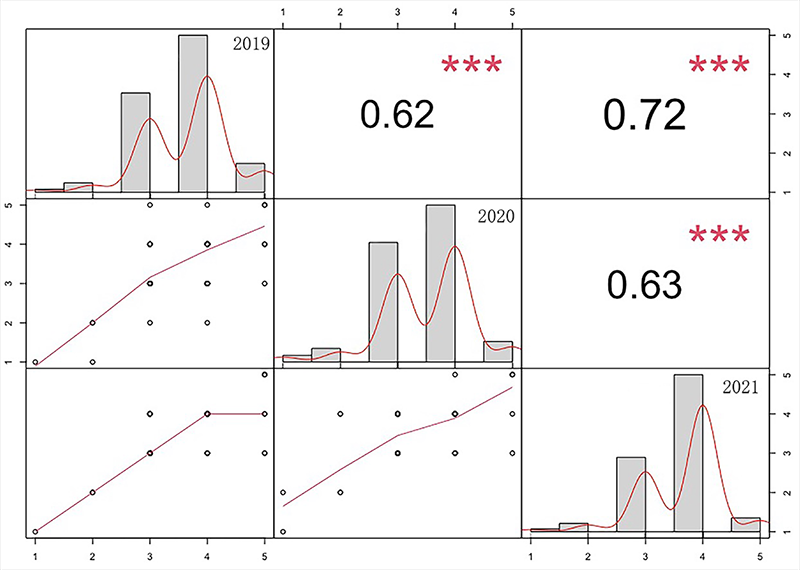


Supplementary Fig1 Pearson correlation coefficients of white rot scores in 3years. ***P < 0.001.


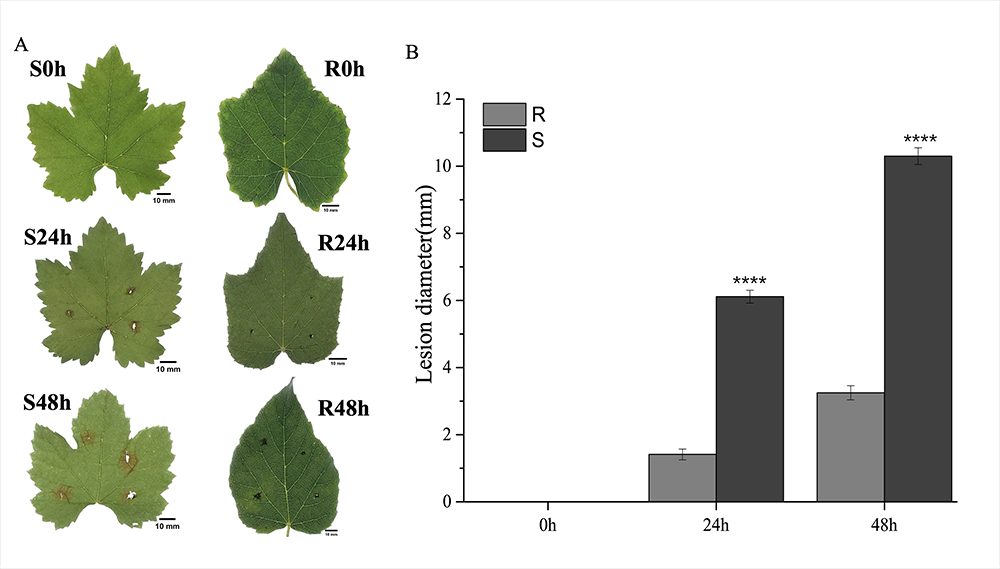


Supplementary Fig2. Lesion area identification of *Vv*MF and *Vd*0940 in response to *C.diplodiella* infection. (A) S and R represent *Vv*MF and *Vd*0940, 24h and 48h represent the hours after infection with *C.diplodiella*. The scale bar is 10 mm. (B) Lesion diameter of infiltrated leaves between *Vv*MF and *Vd*0940 after *C.diplodiella* inoculation at 24 and 48h. Asterisks represent significant differences between the *Vv*MF and *Vd*0940 (****P < 0.0001).


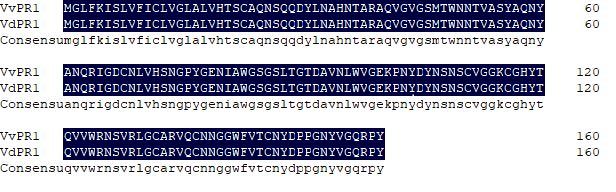


Supplementary Fig3. Alignment of PR1 sequences between *Vv*MF and *Vd*0940 at amino acid levels.


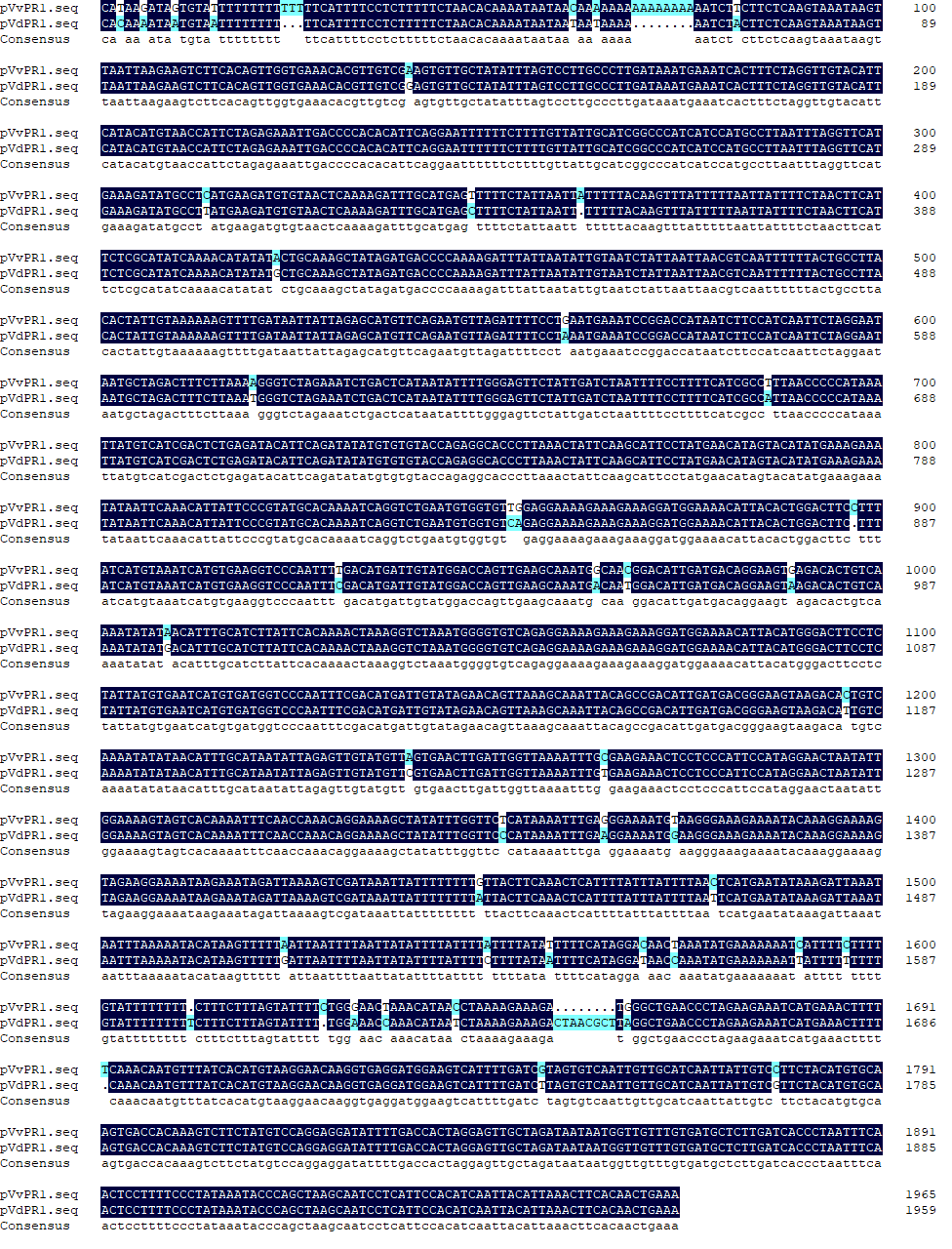


Supplementary Fig4 Alignment of PR1 promoter sequences between *Vv*MF and *Vd*0940


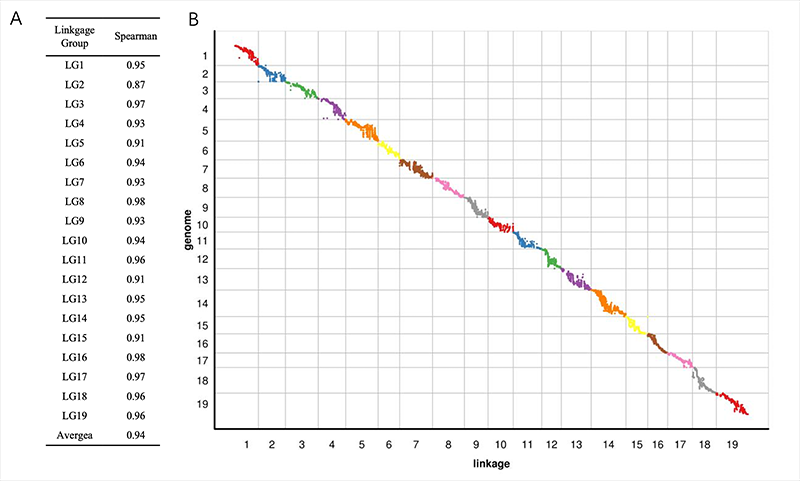


Supplementary Fig5. Collinear analysis of genetic maps and the physical map of the grape genome. A The Spearman correlation coefficients between the genetic and physical positions of each linkage group. B Relationship between genetic and physical positions with each chromosome. The horizontal coordinate is the linkage group, and the vertical coordinate is physical distance.
