# Supplementary material for: QTL detection and candidate gene analysis of grape white rot resistance by interspecific grape (Vitis vinifera L. × Vitis davidii Foex.) crossing
Source: Hortic Res. 2023 Apr 2;10(5):uhad063. doi: 10.1093/hr/uhad063 (PMC10208900; doi:10.1093/hr/uhad063)
Supplement: Web_Material_uhad063 [file web_material_uhad063.zip › Supplementary TableS1. Primers used in this article.docx]

Supplementary Table S1. Primers used in this article

| Primer name | Forward Primer | Reverse Primer |
| --- | --- | --- |
| Vvactin | TTACCGCGGGCAAGAGATAC | ATTCACACGCTCCTTTTGGG |
| PR1CDS | ATGGGGTTGTTTAAGATTTCACTAGTTTTTATTTGTCT | TCAATAAGGACGCTGTCCGACA |
| PR1promoter | CATAAAATAGTGTATTTTTTTTTCCCCATTTTCCTCTTTTTCT | TTTCAGTTGTGAAGTTTAATGTAATTGATGTGGAATG |
| pBI121-VvPR1promoter | GACCATGATTACGCCAAGCTTCATAAGATAGTGTATTTTTTTTTTTTTTCATTTTCC | GGACTGACCACCCGGGGATCCTTTCAGTTGTGAAGTTTAATGTAATTGATG |
| pBI121-VdPR1promoter | GACCATGATTACGCCAAGCTTCACAAAATAATGTAATTTTTTTTTTCATTTTCCT | GGACTGACCACCCGGGGATCCTTTCAGTTGTGAAGTTTAATGTAATTGATG |
| qRTPCR-PR1 | TGGAACAACACCGTAGCCTC | CCAGGCAATGTTCTCCCCAT |
| PR1-no signal peptide | GCTCAAAACTCACAGCAGGACT | TCAATAAGGACGCTGTCCGACA |
| PBI1302-VdPR1 | ACGGGGGACTCTTGACCATGGATGGGGTTGTTTAAGATTTCACTAGTTTTTATTTGTCT | AAGTTCTTCTCCTTTACTAGTATAAGGACGCTGTCCGACA |
