# Supplementary material for: QTL detection and candidate gene analysis of grape white rot resistance by interspecific grape (Vitis vinifera L. × Vitis davidii Foex.) crossing
Source: Hortic Res. 2023 Apr 2;10(5):uhad063. doi: 10.1093/hr/uhad063 (PMC10208900; doi:10.1093/hr/uhad063)
Supplement: Web_Material_uhad063 [file web_material_uhad063.zip › Supplementary TableS2 Main characteristics of linkage groups in female parent 'Manicure Fingerâ_T.docx]

Supplementary TableS2 Main characteristics of linkage groups in female parent 'Manicure Finger’

| Linkage groups | Map of female parent ‘Manicure Finger’ | | | | | | | |  |
| --- | --- | --- | --- | --- | --- | --- | --- | --- | --- |
|  | Genetic distance (cM) | SNP markers | Average  distance (cM) | | | Max Gap (cM) | | Percentage of Gap < 5 (cM) | |
| 1 | 157.3 | 373 | | 0.4 | 26.9 | | 99.2 | |  |
| 2 | 212.7 | 376 | | 0.6 | 55.9 | | 97.9 | |  |
| 3 | 215.7 | 400 | | 0.5 | 17.6 | | 99.8 | |  |
| 4 | 155.6 | 466 | | 0.3 | 7.5 | | 98.7 | |  |
| 5 | 211.0 | 491 | | 0.4 | 9.8 | | 98.8 | |  |
| 6 | 130.1 | 307 | | 0.4 | 9.8 | | 98.0 | |  |
| 7 | 204.4 | 461 | | 0.4 | 26.9 | | 98.9 | |  |
| 8 | 243.8 | 572 | | 0.4 | 6.3 | | 98.6 | |  |
| 9 | 147.3 | 371 | | 0.4 | 8.6 | | 98.7 | |  |
| 10 | 126.2 | 305 | | 0.4 | 7.5 | | 97.0 | |  |
| 11 | 170.8 | 339 | | 0.5 | 28.6 | | 98.2 | |  |
| 12 | 107.4 | 299 | | 0.4 | 19.1 | | 98.0 | |  |
| 13 | 166.8 | 388 | | 0.4 | 22.0 | | 97.7 | |  |
| 14 | 217.6 | 572 | | 0.4 | 8.6 | | 99.0 | |  |
| 15 | 89.2 | 273 | | 0.3 | 9.8 | | 99.3 | |  |
| 16 | 152.0 | 355 | | 0.4 | 14.9 | | 99.7 | |  |
| 17 | 174.7 | 263 | | 0.7 | 36.2 | | 97.7 | |  |
| 18 | 146.9 | 199 | | 0.7 | 53.0 | | 98.0 | |  |
| 19 | 200.0 | 420 | | 0.5 | 9.8 | | 97.6 | |  |
| Total | 3229 | 7230 | | 0.4 | / | | / | |  |
