# Supplementary material for: QTL detection and candidate gene analysis of grape white rot resistance by interspecific grape (Vitis vinifera L. × Vitis davidii Foex.) crossing
Source: Hortic Res. 2023 Apr 2;10(5):uhad063. doi: 10.1093/hr/uhad063 (PMC10208900; doi:10.1093/hr/uhad063)
Supplement: Web_Material_uhad063 [file web_material_uhad063.zip › Supplementary TableS3 Main characteristics of linkage groups in female parent â_~940â_T.docx]

Supplementary TableS3 Main characteristics of linkage groups in female parent ‘940’

| Linkage groups | Map of female parent ‘940’ | | | | | | | |  |
| --- | --- | --- | --- | --- | --- | --- | --- | --- | --- |
|  | Genetic distance (cM) | SNP markers | Average  distance (cM) | | | Max Gap (cM) | | Percentage of Gap < 5 (cM) | |
| 1 | 93.7 | 165 | | 0.6 | 22.0 | | 97.6 | |  |
| 2 | 85.0 | 166 | | 0.5 | 8.6 | | 97.0 | |  |
| 3 | 141.6 | 79 | | 1.8 | 36.2 | | 87.2 | |  |
| 4 | 150.4 | 179 | | 0.8 | 45.1 | | 96.1 | |  |
| 5 | 68.2 | 113 | | 0.6 | 14.9 | | 96.4 | |  |
| 6 | 89.9 | 101 | | 0.9 | 40.4 | | 96.0 | |  |
| 7 | 177.9 | 163 | | 1.1 | 62.4 | | 97.5 | |  |
| 8 | 111.2 | 148 | | 0.8 | 28.6 | | 98.0 | |  |
| 9 | 91.5 | 162 | | 0.6 | 14.9 | | 98.1 | |  |
| 10 | 151.2 | 114 | | 1.3 | 96.6 | | 96.5 | |  |
| 11 | 112.8 | 141 | | 0.8 | 23.6 | | 97.1 | |  |
| 12 | 89.8 | 171 | | 0.5 | 13.6 | | 95.9 | |  |
| 13 | 182.3 | 150 | | 1.2 | 34.2 | | 92.6 | |  |
| 14 | 164.4 | 227 | | 0.7 | 69.8 | | 98.2 | |  |
| 15 | 115.0 | 160 | | 0.7 | 19.1 | | 95.0 | |  |
| 16 | 68.3 | 184 | | 0.4 | 7.5 | | 98.9 | |  |
| 17 | 114.4 | 178 | | 0.6 | 7.5 | | 98.3 | |  |
| 18 | 123.1 | 229 | | 0.5 | 13.6 | | 98.7 | |  |
| 19 | 133.5 | 210 | | 0.6 | 40.4 | | 98.1 | |  |
| Total | 2264 | 3040 | | 0.7 | / | | / | |  |
