# Supplementary material for: QTL detection and candidate gene analysis of grape white rot resistance by interspecific grape (Vitis vinifera L. × Vitis davidii Foex.) crossing
Source: Hortic Res. 2023 Apr 2;10(5):uhad063. doi: 10.1093/hr/uhad063 (PMC10208900; doi:10.1093/hr/uhad063)
Supplement: Web_Material_uhad063 [file web_material_uhad063.zip › Supplementary TableS4 Main characteristics of linkage groups in integrated map .docx]

Supplementary TableS4 Main characteristics of linkage groups in integrated map

| Linkage groups | Integrated map | | | | | | |  |
| --- | --- | --- | --- | --- | --- | --- | --- | --- |
|  | Genetic distance (cM) | SNP markers | Average  distance (cM) | | Max Gap (cM) | | Percentage of Gap < 5 (cM) | |
| 1 | 139.7 | 534 | | 0.3 | 10.3 | 99.6 | |  |
| 2 | 162.1 | 314 | | 0.5 | 11.8 | 99.1 | |  |
| 3 | 197.2 | 463 | | 0.4 | 10.8 | 99.8 | |  |
| 4 | 164.1 | 637 | | 0.3 | 15.9 | 99.5 | |  |
| 5 | 194.6 | 340 | | 0.6 | 8.1 | 99.2 | |  |
| 6 | 129.1 | 406 | | 0.3 | 9.8 | 98.8 | |  |
| 7 | 197.1 | 621 | | 0.3 | 18.1 | 99.4 | |  |
| 8 | 191.3 | 714 | | 0.3 | 17.5 | 99.7 | |  |
| 9 | 141.8 | 529 | | 0.3 | 6.3 | 99.2 | |  |
| 10 | 151.2 | 418 | | 0.4 | 17.2 | 98.3 | |  |
| 11 | 170.8 | 479 | | 0.4 | 17.3 | 98.5 | |  |
| 12 | 117.4 | 465 | | 0.3 | 8.4 | 98.9 | |  |
| 13 | 179.4 | 534 | | 0.3 | 17.2 | 98.3 | |  |
| 14 | 208.5 | 791 | | 0.3 | 6.8 | 99.6 | |  |
| 15 | 130.2 | 428 | | 0.3 | 13.6 | 99.1 | |  |
| 16 | 119.6 | 351 | | 0.3 | 4.3 | 100.0 | |  |
| 17 | 149.2 | 438 | | 0.3 | 5.2 | 99.8 | |  |
| 18 | 143.2 | 423 | | 0.3 | 4.1 | 100.0 | |  |
| 19 | 189.4 | 452 | | 0.4 | 14.8 | 98.7 | |  |
| Total | 3076 | 9337 | | 0.3 | / | / | |  |
