# Supplementary material for: QTL detection and candidate gene analysis of grape white rot resistance by interspecific grape (Vitis vinifera L. × Vitis davidii Foex.) crossing
Source: Hortic Res. 2023 Apr 2;10(5):uhad063. doi: 10.1093/hr/uhad063 (PMC10208900; doi:10.1093/hr/uhad063)
Supplement: Web_Material_uhad063 [file web_material_uhad063.zip › Supplementary TableS5 The QTL for C. diplodiella resistance using both parents map.docx]

Supplementary Table S5 The QTL for *C. diplodiella* resistance using both parents map

| MAP | Year | Chr | LOD threshold^a^ | Peak Lod | Confidence interval | Marker  interval | PVE(%) | Physical  position |
| --- | --- | --- | --- | --- | --- | --- | --- | --- |
| *Vd*0940 | 2019 | 3 | 2.8 | 3.85 | 65.73-67.73 | Marker638599-Marker678198 | 17.4 | 4954300-9416779 |
|  | 2020 | 3 | 2.7 | 4.17 | 58.27-67.73 | marker635032-Marker678198 | 18.6 | 4535710-9416779 |
|  | 2021 | 3 | 2.8 | 3.75 | 66.73-67.73 | Marker651840-Marker678198 | 17 | 6282673-9416779 |
|  |  |  |  |  |  |  |  |  |
| *Vv*MF | 2019 | 3 | 2.8 | 3.48 | 88.308-92.329 | Marker646495-Marker660592 | 15.8 | 5779916-7287503 |
|  |  | 3 | 2.8 | 3.85 | 116.597-118.597 | Marker662747-Maker667716 | 17.4 | 7525855-8114309 |
|  | 2020 | 3 | 2.8 | 3.69 | 85.246-92.329 | Marker644306-Marker660592 | 16.7 | 5572025-7287503 |
|  |  | 3 | 2.8 | 3.98 | 116.597-118.597 | Marker662747-Maker667716 | 17.9 | 7525855-8114309 |
|  | 2021 | 3 | 2.5 | 3.75 | 116.597-118.597 | Marker662747-Maker667716 | 17 | 7525855-8114309 |

^a^ Calculated threshold values using a permutation test at α = 0.05
