# Supplementary material for: QTL detection and candidate gene analysis of grape white rot resistance by interspecific grape (Vitis vinifera L. × Vitis davidii Foex.) crossing
Source: Hortic Res. 2023 Apr 2;10(5):uhad063. doi: 10.1093/hr/uhad063 (PMC10208900; doi:10.1093/hr/uhad063)
Supplement: Web_Material_uhad063 [file web_material_uhad063.zip › Supplementary TableS6. Summary of the QTL for C. diplodiella resistance.docx]

Supplementary TableS6. Summary of the QTL for *C. diplodiella* resistance using integrated map

| Year | LG | LOD threshold^a^ | Confidence interval (cM) | Peak Position(cM) | Peak LOD | PVE (%) |  |
| --- | --- | --- | --- | --- | --- | --- | --- |
|  |  |  |  |  |  |  |  |
| 2019 | 3 | 3.1 | 86.493-86.993 | 86.493 | 3.2 | 14.7 |  |
|  | 3 |  | 91.205-94.568 | 93.205 | 3.49 | 15.9 |  |
|  | 3 |  | 105.077-108.803 | 107.164 | 3.85 | 17.4 |  |
|  | 8 |  | 28.603-30.283 | 28.603 | 3.12 | 14.3 |  |
|  | 12 |  | 73.552-74.444 | 73.684 | 3.3 | 15.1 |  |
|  |  |  |  |  |  |  |  |
| 2020 | 3 | 3.1 | 84.173-94.568 | 87.653 | 4.16 | 18.6 |  |
|  | 3 |  | 105.077-108.803 | 107.164 | 3.98 | 17.9 |  |
|  | 8 |  | 60.295-65.682 | 62.95 | 3.51 | 16 |  |
|  | 8 |  | 71.159-71.935 | 71.159 | 3.81 | 17.2 |  |
|  | 8 |  | 77.418-78.777 | 77.418 | 3.58 | 16.2 |  |
|  | 8 |  | 95.05-96.066 | 96.066 | 3.39 | 15.5 |  |
|  |  |  |  |  |  |  |  |
| 2021 | 3 | 2.9 | 105.596-108.803 | 107.164 | 3.75 | 17 |  |
|  | 18 |  | 69.775-71.43 | 71.43 | 3.7 | 16.1 |  |

^a^ Calculated threshold values using a permutation test at α = 0.05
