# Supplementary material for: QTL detection and candidate gene analysis of grape white rot resistance by interspecific grape (Vitis vinifera L. × Vitis davidii Foex.) crossing
Source: Hortic Res. 2023 Apr 2;10(5):uhad063. doi: 10.1093/hr/uhad063 (PMC10208900; doi:10.1093/hr/uhad063)
Supplement: Web_Material_uhad063 [file web_material_uhad063.zip › Supplementary TableS7 Identification of markers co-segregated with resistance to C.diplodiella using the Kruskalâ_"Wallis algorithm.docx]

Supplementary TableS7 Identification of markers co-segregated with resistance to *C.diplodiella* using the Kruskal–Wallis algorithm

| Locus | LG | Df^a^ | Significance level | | |
| --- | --- | --- | --- | --- | --- |
|  |  |  | 2019 | 2020 | 2021 |
| Marker655251 | 3 | 1 | *** | **** | ** |
| Marker656426 | 3 | 1 | *** | **** | ** |
| Marker663881 | 3 | 1 | **** | **** | ** |
| Marker651840 | 3 | 1 | ** | ** | ** |
| Marker651917 | 3 | 1 | ** | ** | ** |
| Marker653959 | 3 | 1 | ** | ** | ** |
| Marker661751 | 3 | 1 | ** | ** | ** |
| Marker662332 | 3 | 1 | ** | ** | ** |
| Marker664145 | 3 | 1 | ** | ** | ** |
| Marker664326 | 3 | 1 | ** | ** | ** |
| Marker665083 | 3 | 1 | ** | ** | ** |
| Marker666805 | 3 | 1 | ** | ** | ** |
| Marker667643 | 3 | 1 | ** | ** | ** |
| Marker668176 | 3 | 1 | ** | ** | ** |
| Marker668230 | 3 | 1 | ** | ** | ** |
| Marker670111 | 3 | 1 | ** | ** | ** |
| Marker670940 | 3 | 1 | ** | ** | ** |
| Marker667716 | 3 | 1 | *** | *** | ** |
| Marker672442 | 3 | 1 | ** | ** | ** |
| Marker673704 | 3 | 1 | ** | ** | ** |
| Marker676005 | 3 | 1 | ** | ** | ** |
| Marker678198 | 3 | 1 | ** | ** | ** |

*P = **0.01, ***0.001, ****0.0001. ^a^Degrees of freedom
