# Supplementary figures and images for: QTL detection and candidate gene analysis of grape white rot resistance by interspecific grape (Vitis vinifera L. × Vitis davidii Foex.) crossing
Source: Hortic Res. 2023 Apr 2;10(5):uhad063. doi: 10.1093/hr/uhad063 (PMC10208900; doi:10.1093/hr/uhad063)

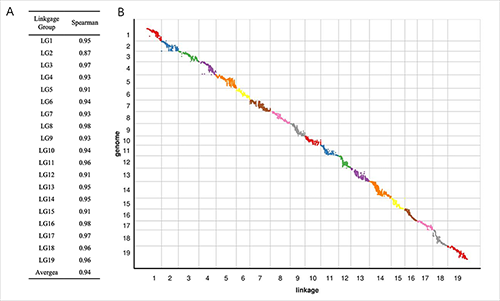

Supplement: Web_Material_uhad063 [file web_material_uhad063.zip › 300 Supplementary Fig5. Collinear analysis of genetic maps and the physical map of the grape genome.tif]

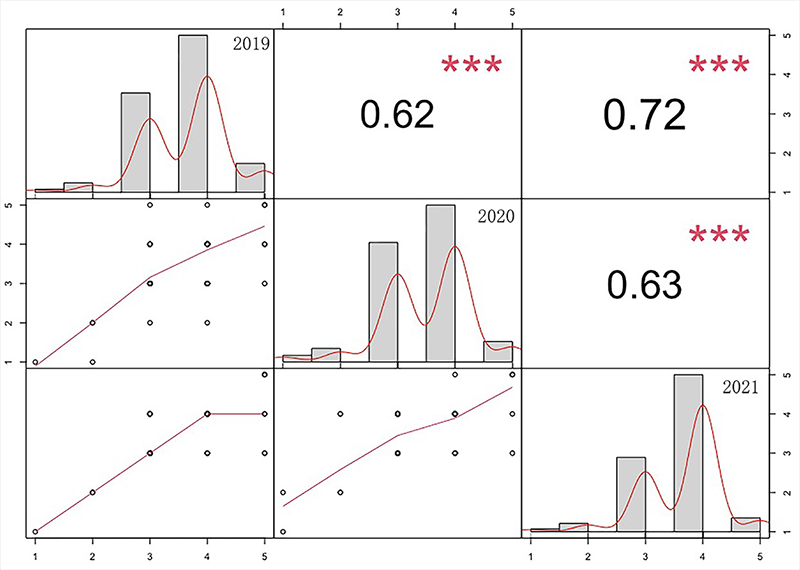

Supplement: Web_Material_uhad063 [file web_material_uhad063.zip › Supplementary Fig1 Pearson correlation coefficients of white rot scores in 3years.tif]

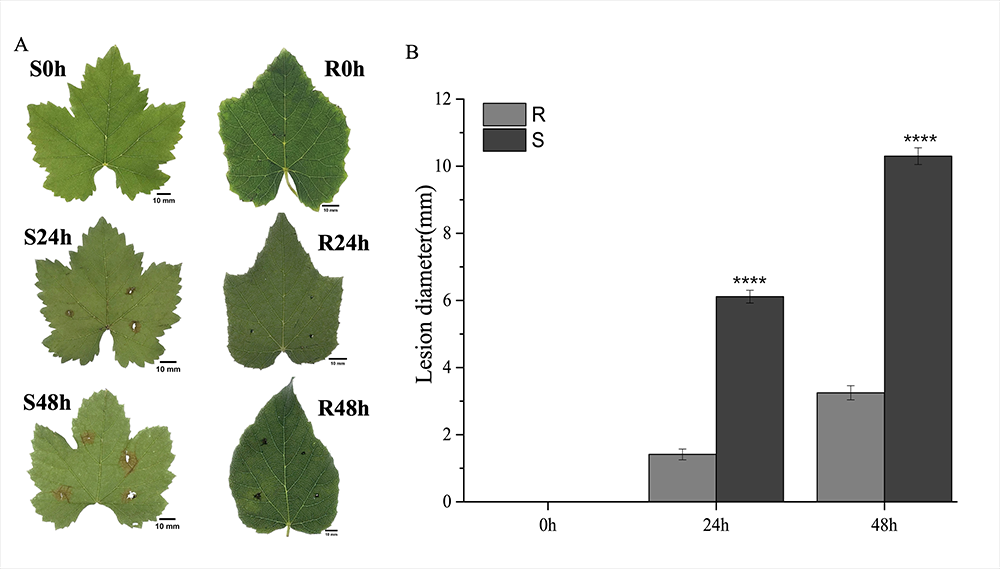

Supplement: Web_Material_uhad063 [file web_material_uhad063.zip › Supplementary Fig2. Lesion area identification of VvMF and Vd0940 in response to C.diplodiella infection.tif]

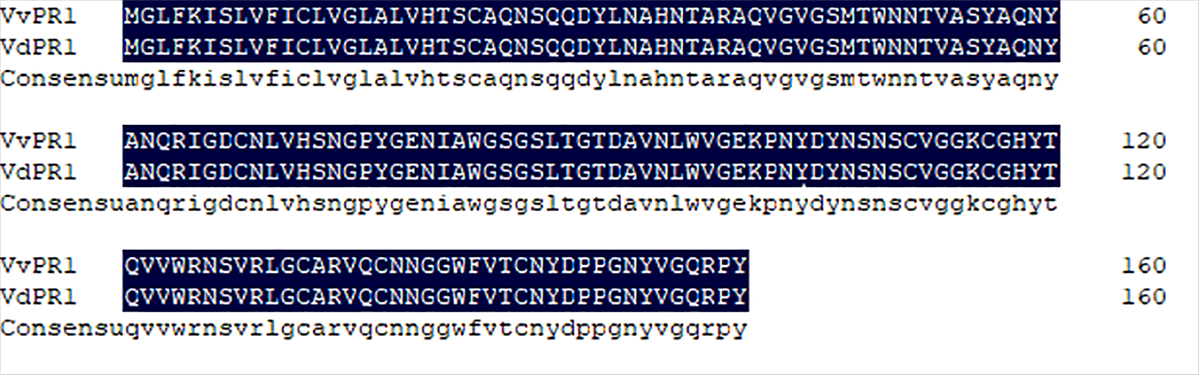

Supplement: Web_Material_uhad063 [file web_material_uhad063.zip › Supplementary Fig3. Alignment of PR1 sequences between VvMF and Vd0940 at amino acid levels.tif]

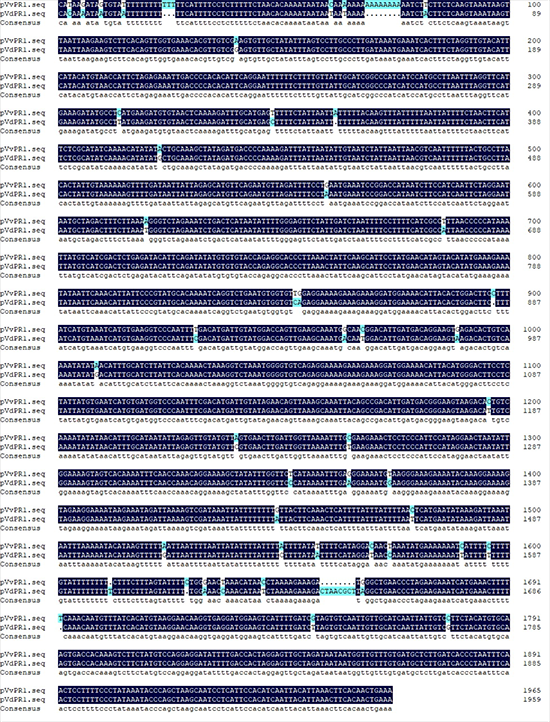

Supplement: Web_Material_uhad063 [file web_material_uhad063.zip › Supplementary Fig4 Alignment of PR1 promoter sequences between VvMF and Vd0940.tif]
